# Prediction of the Clinical Course of Immune Thrombocytopenia in Children by Platelet Kinetics

**DOI:** 10.1097/HS9.0000000000000960

**Published:** 2023-10-27

**Authors:** Julien Lejeune, Violette Raoult, Mathilde Dubrasquet, Romane Chauvin, Coralie Mallebranche, Isabelle Pellier, Françoise Monceaux, Sophie Bayart, Audrey Grain, Emmanuel Gyan, Noémie Ravalet, Olivier Herault, David Ternant

**Affiliations:** 1 Pediatric Onco-Hematology Unit, CHU de Tours, France; 2 CNRS ERL7001, EA 7501 GICC, University of Tours, France; 3 Pediatric Immuno-Hemato-Oncology Unit, CHU Angers, France; 4 Pediatric Unit, CH Orléans, France; 5 Pediatric and Adolescent Unit, CHRU de Rennes, France; 6 Pediatric Immuno-Hemato-Oncology Unit, CHU Nantes, France; 7 Department of Biological Hematology, Tours University Hospital, Tours, France; 8 EA 7501 « Transplantation, Immunology, Inflammation », University of Tours, France

## Abstract

Childhood immune thrombocytopenia (ITP) is a rare autoimmune disorder characterized by isolated thrombocytopenia. Prolonged ITP (persistent and chronic) leads to a reduced quality of life for children in many domains. To provide optimal support for children, with ITP, it is important to be able to predict those who will develop prolonged ITP. This study aimed to develop a mathematical model based on platelet recovery that allows the early prediction of prolonged ITP. In this retrospective study, we used platelet counts from the 6 months following the diagnosis of ITP to model the kinetics of change in platelet count using a pharmacokinetic–pharmacodynamic model. In a learning set (n = 103), platelet counts were satisfactorily described by our kinetic model. The K_heal_ parameter, which describes spontaneous platelet recovery, allowed a distinction between acute and prolonged ITP with an area under the curve (AUC) of 0.74. In a validation set (n = 58), spontaneous platelet recovery was robustly predicted using platelet counts from 15 (AUC = 0.76) or 30 (AUC = 0.82) days after ITP diagnosis. In our model, platelet recovery quantified using the k_heal_ parameter allowed prediction of the clinical course of ITP. Future prospective studies are needed to improve the predictivity of this model, in particular, by combining it with the predictive scores previously reported in the literature.

## INTRODUCTION

Childhood immune thrombocytopenia (ITP) is a rare hematological disorder that occurs in approximately 5 to 10 children per 100,000.^1^ It is an acquired autoimmune disorder characterized by isolated thrombocytopenia (peripheral blood platelet count <100 × 10^9^/L) caused by increased platelet destruction, alterations in cellular immunity, and impaired platelet production.^2^ ITP is a heterogeneous disease that shows variable etiology, bleeding symptoms, need for treatment, response to therapy, and duration.^3^ There are several different types of ITP duration in children: (1) acute ITP, lasting not longer than 3 months, (2) persistent ITP, which covers a period between 3 and 12 months after diagnosis, and (3) chronic ITP, with more than 12 months of duration.^4^

The duration of ITP is difficult to predict. Generally, the longer it persists, the heavier the disease burden for the children and their families. On a daily basis, ITP leads to a reduced quality of life of children in many domains, especially for persistent and chronic ITP.^5^ For example, severe daily fatigue is reported by children with chronic ITP.^6,7^ Chronic ITP also affects children’s social lives, with restrictions on certain physical activities and absenteeism from school to attend medical appointments or have blood tests.^8,9^ At the psychological level, chronic ITP is associated with increased levels of stress, anxiety, depression, and isolation in children.^10^

In an effort to provide optimal support to children and their families, a number of studies have examined the factors associated with the clinical course of ITP. Among the many clinical predictors studied, being female, being older (age ≥11 years), and having had an insidious onset of the disease, without infectious or vaccinal triggers, have been associated with chronic ITP.^11^

Among the biological factors tested, only the presence of antinuclear antibodies and the platelet count at diagnosis have been shown to be associated with the clinical course of ITP.^11^ At diagnosis, non-severe thrombocytopenia (>20 × 10^9^/L) is also associated with a risk of chronic ITP,^11^ whereas severe thrombocytopenia (<5 × 10^9^/L) is associated with a low risk of chronicity.^12^ Further, Schmidt et al developed a score that gathered these factors into a recovery score displaying good performance to predict a prolonged disease for a given patient.^13^ However, their model does not account for platelet duration over time. To date, few studies have precisely investigated the evolution of platelet count kinetics and the risk of chronicity of ITP. Choi et al found that patients with chronic ITP had significantly lower platelet counts 1 and 3 months after diagnosis than patients with acute ITP.^14^ On the contrary, Higashide et al found no association between ITP progression and platelet counts at 12 months.^15^

In this context, we aimed to develop a mathematical model based on platelet kinetics that allows the early prediction of ITP persistence or chronicity.

## METHODS

### Data collection

#### Patient population

This retrospective observational study was carried out using data from the medical records of patients under 18 years of age followed for ITP in 5 French hospitals (Tours, Orléans, Nantes, Angers, Rennes). The diagnosis of ITP was made according to the 2009 international recommendations.^16^ It is a diagnosis of elimination made when there is a combination of clinical hemorrhagic syndrome (purpura, mucosal, or visceral hemorrhage) and isolated peripheral thrombocytopenia ≤100 × 10^9^/L. Thrombocytopenia should not be associated with other types of cytopenia, immune deficiency, or coagulation disorders. The presence of purpura should not be secondary to hematological malignancy, cancer, alloimmunization, or medication. A relapse is characterized by the recurrence of thrombocytopenia ≤100 × 10^9^/L, with or without a clinical bleeding syndrome.^16^ ITP was considered to be acute when the thrombocytopenia improved within 3 months, persistent when the recovery from thrombocytopenia took between 3 and 12 months, and chronic when thrombocytopenia persisted beyond 12 months.^16^ Prolonged ITP was considered to be the association of persistent and chronic ITP. This study was approved by the health data club of the Tours Hospitals (F20200907070428) and by the clinical research assistance ethics group (#2020 076).

#### Enrollment criteria

Patients aged 0–18 years with ITP followed at the participating hospitals were assessed. Secondary ITP or ITP with associated hematological or extra-hematological syndromes (hematological malignancy, acquired or primary immunodeficiency, genetic syndrome, systemic lupus erythematosus) were excluded. Patients who received a consultation or hospitalization related to ITP but whose follow-up was carried out in another center and those with missing data (less than 5 platelet counts within the first 90 days) were not assessed.

#### Data collection

The following clinical data were collected from the patient's medical records: age at diagnosis, gender, sex, Buchanan score,^17^ acute or insidious presentation (bleeding symptoms for more than 14 days before diagnosis), and recent infections or vaccination within 21 days before the diagnosis of ITP. The treatment received and the dates of administration, as well as other medical treatment, during the first 6 months were recorded. Patients in the study were treated in the acute phase according to French recommendations:^18^ in the presence of thrombocytopenia <10 × 10^9^/L or a bleeding syndrome with a Buchanan score ≥3, treatment consisted of an injection of polyvalent immunoglobulins (0.8–1 g/kg) or 4 mg/kg/day of prednisolone/prednisone for 4 days. If one of these treatments is ineffective, it is possible to combine them simultaneously. In all other cases, a watchful waiting strategy was implemented. In case of relapse, either a combination of corticosteroid and polyvalent immunoglobulins can be used. Biological data collected consisted of the platelet and leukocyte counts at diagnosis, evolution of platelet counts in the first 6 months, immunoglobulin G levels, and the presence of anti-nucleus antibodies.

### Statistical analysis

#### Population kinetic modeling of platelet counts

The aim of this study was to provide an early prediction of disease progression, i.e., prolonged (persistent/chronic) versus acute ITP. Platelet recovery following treatment was assessed using a kinetic model that was built, validated and which predictive performance was challenged in an external validation dataset. This model, derived from pharmacokinetic and pharmacokinetic–pharmacodynamic (PK-PD) models,^19,20^ was previously used to describe platelet time course^21^ and allowed a description of the platelet count over time and is presented in detail in Supporting Information Material. Briefly, the model described the influence of treatment on platelet count turnover and included a parameter (k_heal_) that describes spontaneous platelet recovery and, thus, ITP improvement (Supporting Information Material, Figs. S1 and S2). This parameter describes a zero-order rate of “natural” platelet recovery; therefore, the more the k_heal_ value, the faster the platelet recovery. Initial application of the model showed that this value was 70-fold fold lower in persistent/chronic than acute disease (*P* = 0.002, Supporting Information Material). This motivated us to investigate the potential use of the k_heal_ value estimated for each patient as an early predictor of the possible evolution toward persistence/chronicity.

#### Data splitting

To ensure the predictive performance of a model, different datasets must be used to estimate the kinetic parameters (learning phase) and evaluate model-predicted disease evolution (validation phase). Thus, learning and validation subsets were made using the data for patients from 3 (Tours, Orléans, and Nantes) and 2 centers (Angers and Rennes), respectively (Supporting Information Material, Fig. S1). We evaluated the predictive performance of the model by evaluating the full validation subset (Vfull), as well as 2 subsets for which the validation subset consisted of platelet counts truncated to 30 (TV30) or 15 (TV15) days. The full validation subset was made to validate the descriptive performance of the model whereas the truncated validation subsets were made to validate the predictive performance of the model in predicting patient status using respectively 30 days and 15 days after diagnosis.

#### Model development

Kinetic model parameters were estimated by nonlinear mixed-effect modeling^20^ (population approach) using Monolix Suite 2019 (Lixoft, Antony, France). With this approach, widely used in pharmacology, data from patients of a given population are simultaneously computed to estimate the interindividual distribution of kinetic parameters. This interindividual distribution allows quantification of: (1) the mean (typical) value of each parameter, (2) interindividual variability (interindividual variance), (3) the influence of individual sources on such variability (covariates), and (4) the estimated parameter values for each patient.

The learning subset was used for the development of our platelet kinetic model and to estimate the interindividual distribution of the kinetic parameters and then individual parameter values. Individual k_heal_ estimates of patients from the learning subset were used to determine a threshold value for which a k_heal_ below or above this threshold is predictive of persistent/chronic or acute disease, respectively. The value of the k_heal_ threshold was determined using receiver-operating characteristic (ROC) analysis, with respect to the actual diagnosis, by minimizing the Youden index (defined as Y = sensitivity + specificity − 1).

#### Evaluation of the predictive performance of the model

These distributions were then applied to truncated validation subsets TV30 and TV15 to estimate the respective individual kinetic parameter values. Then individual k_heal_ estimates from Vfull, TV30, and TV15 were tested to predict disease duration. These estimates were compared to the threshold value determined in the learning subset: patients with k_heal_ estimates below or above the threshold were inferred as acute or persistent/chronic ITP. Finally, these inferences were compared to the actual diagnosis by calculating the sensitivity (Se), specificity (Sp), positive (PPV), and negative (NPV) predictive values, and Cohen’s kappa (Ka).

## RESULTS

### Patient selection and characterization

**Figure 1.**
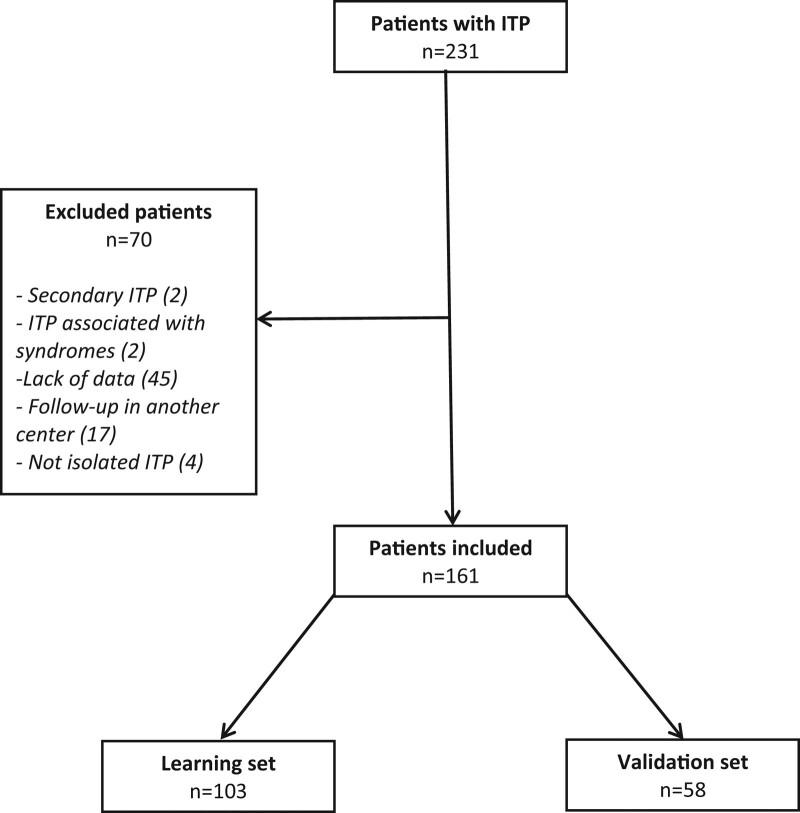
**Flowchart of the selection of patients with ITP.** ITP = immune thrombocytopenia.

We identified 231 patients who consulted for ITP at the study hospitals. Seventy patients were excluded because: they did not have isolated ITP (n = 4), due to a lack of data (n = 45), they were followed-up in another hospital (n = 17), they had ITP associated with syndromes (n = 2) or secondary ITP (n = 2 patients with presence of antinuclear antibody) (Fig. 1). Finally, 161 patients were included in our study, allowing us to constitute a learning cohort of 103 patients and an external validation cohort of 58 patients (Table 1). The learning cohort consisted of 68 patients with acute ITP, 11 with persistent ITP, and 24 with chronic ITP. The external validation cohort consisted of 35 patients with acute ITP, 3 with persistent ITP, and 20 with chronic ITP. No significant difference was found between the various clinical and biological characteristics of these 2 cohorts (Table 1).

**Table 1:** Clinical and Biological Characteristics of Children With ITP in the Learning and Validation Datasets

	Learning set, n = 103 (%)	Validation set, n = 58 (%)	*P*
• ITP			
Acute	68 (66)	35 (60.3)	0.47
Persistent	11 (10.7)	3 (5.2)	
Chronic	24 (23.3)	20 (34.5)	
• Sex			
Boys	46 (44.6)	27 (46.5)	0.81
Girls	57 (55.4)	31 (53.5)	
• Age at diagnosis			
Average (yrs)	5.27	6.14	0.21
0–23 mo	27 (26.2)	8 (13.7)	0.06
24 mo–5.9 yrs	36 (34.9)	22 (38)	0.7
6-10.9 yrs	24 (23.3)	20 (34.5)	0.48
≥11 yrs	16 (15.6)	8 (13.7)	0.45
• Buchanan score			
0–2	53 (51.4)	34 (58.6)	0.76
≥3	50 (48.6)	24 (41.4)	
Preceding infection or vaccination	55 (53.4)	23 (39.6)	0.09
Sudden onset	45 (43.6)	21 (36.2)	0.35
Platelet count <10 × 10^9^/L at diagnosis	70 (68)	39 (67.2)	0.92

ITP = immune thrombocytopenia.

During the first 3 months of duration, no significant difference was found between the learning and validation sets for patients who received either corticosteroids or polyvalent immunoglobulins (Table 2). In contrast, a higher proportion of patients received an association of corticosteroid and polyvalent immunoglobulins in the validation set (*P* = 0.001) (Table 2).

**Table 2:** Treatments Received in the First 3 Months of Duration in the Learning and Validation Sets

	Learning Set (n = 103)			Validation Set (n = 58)				
	Acute ITP, n = 68 (%)	Prolonged ITP		Acute ITP, n = 35 (%)	Prolonged ITP			*P*
		Persistent, n = 11 (%)	Chronic, n = 24 (%)		Persistent, n = 3 (%)		Chronic, n = 20 (%)	
Watchful waiting strategy	3 (2.9)	1 (0.9)	0 (0)	5 (8.6)	0 (0)		1 (1.7)	
Total	4 (3.8)			6 (10.3)				0.1
Corticosteroids only								
1 cure	8 (7.9)	0 (0)	2 (1.9)	6 (10.3)	1 (1.7)		4 (6.9)	
2 cures	1 (0.9)	0 (0)	2 (1.9)	0 (0)	0 (0)		1 (1.7)	
3 cures	4 (3.9)	1 (0.9)	3 (2.9)	0 (0)	0 (0)		2 (3.4)	
Total	21 (20.3)			14 (24)				0.6
Polyvalent immnunoglobulins only								
1 cure	25 (24.6)	2 (1.9)	2 (1.9)	9 (15.7)		0 (0)	1 (1.7)	
2 cures	13 (12.6)	3 (2.9)	2 (1.9)	1 (1.7)		0 (0)	1 (1.7)	
3 cures	2 (1.9)	3 (2.9)	6 (5.7)	0 (0)		0 (0)	1 (1.7)	
Total	58 (56.3)			13 (22.5)				0.8
Corticosteroids and polyvalent immunoglobulins	12 (11.8)	1 (0.9)	7 (6.9)	14 (24.1)		2 (3.4)	9 (15.7)	
Total	20 (19.6)			25 (43.2)				0.001

ITP = immune thrombocytopenia.

### Prediction of ITP evolution

From the learning set, platelet counts were satisfactorily described by our kinetic model, and the kinetic parameters were accurately estimated (Supplementary Material, Figs. S3 and S4, Tables S1 and S2 ). Notably, the mean k_heal_ value was 0.0012 day^-1^, with very high interindividual variability (290%); such variability was responsible for most of the variability of the platelet count data over time (Supporting Information Material). ROC analysis showed a threshold value of k_heal_ = 0.00743 days; patients with a k_heal_ below this value were declared to have persistent/chronic disease, with a Se of 0.72, Sp of 0.66, a PPV of 0.53, a NPV of 0.81, and Ka of 0.68 (Table 3).

**Table 3:** Prediction of Clinical Course (Acute vs Prolonged ITP) in the Learning and Validation Datasets

Parameter	Learning set	Vfull	TV30	TV15
Sensitivity	0.72	0.70	0.70	0.57
Specificity	0.66	0.75	0.75	0.81
PPV	0.53	0.64	0.64	0.65
NPV	0.81	0.79	0.79	0.74
Kappa	0.68	0.73	0.73	0.71
AUC	0.74	0.82	0.82	0.76
Acute matches	26	16	16	13
Prolonged matches	44	26	26	28
Acute mismatches	23	9	9	7
Prolonged mismatches	10	7	7	10

Predictive parameters were determinated in the learning and validation set with platelets of the first 6 months (Vfull) and of the 30 (TV30) or 15 (TV15) days of clinical duration.

AUC = area under the receiver operative characteristic (ROC) curve; NPV = negative predictive value; PPV = positive predictive value; TV = validation subset truncated of platelet counts 30 or 15 days after diagnosis.

Use of the interindividual distribution of the kinetic parameters and threshold determined in the learning set on the validation set yielded similar values (Se = 0.70, Sp = 0.75, PPV = 0.64, NPV = 0.79, Ka = 0.73) (Table 3). These values remained similar using validation sets with platelet counts truncated to 30 and 15 days, except for Se, which was lower for 15 days (0.57, Table 2). The k_heal_ value was not associated with the clinical-biological characteristics (age at diagnosis, sex, insidious onset of the ITP, infectious or vaccinal triggers, platelet count at diagnosis) of the children with ITP in either the learning or validation sets.

## DISCUSSION

We report a mathematical model that describes the evolution of platelet counts over time and allows early prediction of prolonged ITP in children.

Early prediction of prolonged ITP has already been reported in previous works. Besides, previous publications used linear regression to describe platelet count evolution over time but reported conflicting results, this evolution being related^14^ or not^15^ with disease evolution.

In this work, we report the first kinetic model quantifying the kinetics of platelet count over time in ITP children. This model is made of a differential equation system describing platelet turnover and accounts for the disease onset and evolution, and of treatment.^22^ Model parameters were estimated as nonlinear mixed-effect modeling (population approach). The population approach has been extensively used to describe pharmacokinetic and PK–PD relationship of drugs, biomarker kinetics,^23,24^ and tumor growth^25^ over time. Notably, this approach was used to describe the kinetics of blood cell lines after myeloablative chemotherapy.^19^ One of the main advantages of population modeling is that it does not require a large number of subjects. The increase in the use of population kinetic modeling may open the way to the modeling of complex phenomena, such as the response to treatment or prediction of the clinical course.

Interestingly, our model allows quantifying the natural disease recovery by the parameter k_heal_, which presents a very large interindividual variability. This value may be interpreted as the natural recovery if no treatment is administered: platelet recovery is slower in patients with a lower k_heal_ value. Being a first-order rate constant, a «half-life» of recovery may be derived by calculating T½-heal = ln(2)/k_heal_ = 1.6 years in mean in the learning cohort. Early attempts showed that k_heal_ is strongly associated with prolonged disease (p = 1.02. 10^-8^, Supporting Information Material). Half-life of recovery was therefore 6 months and 35 years in acute and prolonged PTI patients, respectively. However, this value should be considered with caution since was estimated from treated patients.

Our model showed good performance in early discrimination between acute versus prolonged ITP starting from 15 days after diagnosis. The predictive performance of our model is similar to the clinical score developed by Schmidt et al, with comparable ROC area under the curve of approximately 0.70.^13^ Schmidt ITP recovery score was calculated in patients of our validation subset and showed higher sensitivity (0.83 versus 0.70) but lower specificity (0.65 versus 0.75) than our kinetic model (Supporting Information Material, Table S3). We believe our approach to be complementary to the score developed by Schmidt et al; our approaches may be gathered which might improve the prediction of prolonged disease (Supporting Information Material, Table S4).

Nevertheless, our model was developed in a retrospective observational data of a relatively small number of treated patients (n = 103). There may be a time-dependence in these data and differences in monitoring habits of each center might have induced bias in model parameter estimation. Therefore, this may have led to several limitations. First, even if trends were observed, the relatively limited number of patients hampered us from an extensive description of the interindividual distribution of k_heal_ parameter. Indeed, we were not able to detect sufficiently significant association of k_heal_ with demographic (age, gender) or biological characteristics (eg, previous vaccination and/or infection). Second, our model was not able to discriminate persistent versus chronic ITP. Third, since our data had only a few control patients (watchful waiting strategy), our model might not be extrapolated outside its scope, i.e. ITP children treated with polyvalent immunoglobulins or corticosteroids. Notably, scenarios concerning untreated patients might not be tested using our model. These limitations may be overcome by refining kinetic model parameter estimates on prospective data, in which all patients would be monitored in the same way (same number of platelet counts), regardless of the treatment or evolution of ITP, will be necessary.

In conclusion, we developed the first kinetic model of platelet course that accounts for platelet destruction and recovery in ITP children. Our model is suitable for predicting disease evolution (acute versus persistent/chronic). Our model will be improved by refining parameter distribution estimates in new prospective studies.

## ACKNOWLEDGMENTS

The authors would like to thank all the hospitals that participated in this study.

## AUTHOR CONTRIBUTIONS

JL, EG, NR, and OH conceived the study and JL wrote the manuscript. VR, MD, CM, IP, FM, SB, and AG provided study data. DT conducted all data analyses. All authors read and approved the final version of the manuscript.

## DISCLOSURES

The authors have no conflicts of interest to disclose.

## SOURCES OF FUNDING

The authors declare no relevant sources of funding for this manuscript.

## Supplementary Material
