# Supplementary material for: Prediction of the Clinical Course of Immune Thrombocytopenia in Children by Platelet Kinetics
Source: Hemasphere. 2023 Oct 27;7(11):e960. doi: 10.1097/HS9.0000000000000960 (PMC10615561; doi:10.1097/HS9.0000000000000960)
Supplement: Supplementary file 1 [file hs9-7-e960-s001.docx]

# Supplementary file for Can platelet kinetics predict the clinical course of immune thrombocytopenia in children?

# Supplemental methods

Platelet count data were analyzed using kinetic models derived from compartmental models commonly used to describe pharmacokinetic and pharmacokinetic-pharmacodynamic data. Kinetic models are derived from dynamical models written as systems of differential equations. The interindividual distribution of model parameters was estimated using nonlinear mixed-effects modeling (population approach).

**Figure S1.** Flow chart of kinetic model development, validation and challenge

## Structural model

Our platelet count kinetic model was made on the basis of Friberg transit model, which has been commonly used to describe hematologic cell evolution over time (1), including platelet time course (2). These models are characterized by sequential compartments representing the different cell maturation between progenitors and mature circulating cells (figure 2). In this study, progenitors are assumed to destroy circulating cells.


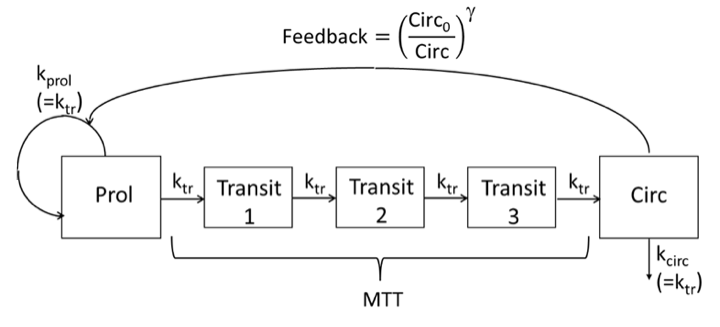


**Figure S2.** Platelet maturation described as a Friberg model (1). Legends: Prol, progenitor compartment; transit: compartment describing maturation states (three compartments are assumed in Friberg models); C_irc_: circulating mature cells; k_prol_: progenitor first-order proliferation rate constant; k_tr_: first-order transit rate constant; k_circ_: first-order mature cell output rate constant; MTT: mean transit time, which is the time for a given cell for progenitor-mature cell maturation. For parameter estimation, Friberg models assume k_prol_ = k_tr_ = k_circ_ (2). The term of feedback assumes a negative regulation of progenitor proliferation by mature circulating cells.

Friberg model allow quantifying progenitor cell proliferation (Prol). This proliferation (k_prol_) is assumed to be negatively impacted $\left( \frac{\mathrm{Circ}_{0}}{\mathrm{Circ}} \right)$by circulating cell count; this negative feedback is stronger for higher mature cell counts and vice-versa. Cell maturation is described using transfer rate constant (k_tr_) across the three transit compartments towards mature cells (Circ), which undergo endogenous output (k_circ_). Platelet elimination by auto-antibodies triggers thrombopenia, which releases from negative feedback, thus leads to a re-increase of progenitor proliferation and a (delayed) mature cell recovery. Friberg model is written as the following system of differential equations:

$$\frac{\mathrm{dProl}}{\mathrm{dt}}=k_{\mathrm{prol}}. Prol. {\left( 1-E_{\max} . \frac{C}{\mathrm{IC}_{50}+C} \right). \left( \frac{\mathrm{Circ}_{0}}{\mathrm{Circ}} \right)}-k_{\mathrm{tr}} . Prol$$

$$\frac{dTransit1}{\mathrm{dt}}=k_{\mathrm{tr}} . Prol-k_{\mathrm{tr}} . Transit1$$

$$\frac{dTransit2}{\mathrm{dt}}=k_{\mathrm{tr}} . Transit1-k_{\mathrm{tr}} . Transit2$$

$$\frac{dTransit3}{\mathrm{dt}}=k_{\mathrm{tr}} . Transit2-k_{\mathrm{tr}} . Transit3$$

$$\frac{\mathrm{dCirc}}{\mathrm{dt}}=k_{\mathrm{tr}} . Transit3-f\left( k_{circ} \right). Circ$$

This model has 5 compartments: Prol, Transit(n=3) and Circ, which are cell counts in progenitor, transit and mature cell compartments, respectively, where Circ_0_ is baseline platelet count (before disease onset), first-order k_prol_, k_tr_ and k_circ_ are first-order constants of progenitor proliferation, maturation and mature cell output, respectively and γ is negative feedback exponent. Friberg modeling assumes k_prol_ = k_tr_ = k_circ_ (1). The delay between progenitor input and mature cell maturation is quantified using the mean transit time (MTT) parameter which depends on the number of transit compartments (n) and k_tr_, as $MTT=\frac{(n+1)}{k_{\mathrm{tr}}}$

In addition, *f*(k_circ_) function describes the modulation of auto-antibody-induced platelet destruction. This modulation is written as the following direct Emax model:

$f\left( k_{\mathrm{circ}} \right)=K_{\mathrm{MAX}}-\left( K_{\mathrm{MAX}}-k_{\mathrm{circ}} \right).\left( \frac{G}{G_{50}+G} \right)$

where K_MAX_ is maximum disease-induced platelet destruction, G is the function describing platelet recovery over time and G_50_ is the value of G leading to a decrease of K_MAX_ of 50%. Of note, k_circ_ is the minimum value of f(k_circ_) interpreted as the “physiologic” endogenous platelet elimination. The G function is written as follows:

$$\frac{\mathrm{dG}}{\mathrm{dt}}=k_{\mathrm{heal}}-k_{\mathrm{el}} . G$$

Where k_el_ is first-order disease persistence rate constant and k_heal_ is zero-order spontaneous disease recovery rate constant. Treatment of PTI was assumed to increase the value of G function. Disease recovery depends not only on treatment efficacy, but also to spontaneous platelet recovery (k_heal_). Disease recovery is faster as k_heal_ is high. Therefore, k_heal_ is considered as the parameter of interest, its association with prolonged disease was especially investigated.

## Parameter estimation

Our model necessitated to estimate seven parameters, i.e. kheal, Circ0, MTT, γ, KMAX, G50 and kel. This estimation was made using nonlinear mixed-effect modeling (population approach). This approach consists in describing interindividual distribution of the seven parameters in a given patient population by estimating mean and interindiviual variance of each of them. However, even if mean value of all model parameters is estimable, this is usually not the case for interinvidual variances; their value is fixed to 0 if not sufficiently accurate. In addition,

Population modelling (2,3) has been used to describe pharmacokinetic data since the early 70’s. If individual modelling is used to estimate the parameters of interest (e.g. the drug clearance in pharmacokinetics) at the “individual level”, i.e. for each individual taken separately from the others, the basic principle of population modelling is to estimate parameters at the “population level”15. The main goals of population modelling are to determine the distribution of the values of the parameters of interest in a population and to quantify the influence of the individual sources of variability in this population. Using a population approach, data from all individuals in a given population are computed simultaneously to estimate the interindividual distribution of parameters of interest. This interindividual distribution allows the quantification of (i) the “mean” (referred as “typical”) value of each parameter, (ii) the interindividual variability (referred as “interindividual variance”). In addition, population approach allows not only testing, but also quantifying the influence of individual sources of variability (“covariates”). This test is made as in most of multivariate statistical analyses. Each model has an associated objective function (OF) value; the lower the OF, the better the data fitting. The OF (most often -2 log-likelihood) is used to compare two nested models; for which the difference in OF follows a χ^2^ distribution.

## Statistical models

Statistical models allow describing interindividual variability and residual error (2,3).

*Interindividual model.* Pharmacokinetic parameters were assumed to follow a lognormal law, interindividual model was therefore exponential i.e. θ_i_ = θ_TV_ . exp(η_i_), where θ_i_ is the estimated individual parameter, θ_TV_ is the typical value of the parameter and η_i_ is the random effect for the i^th^ patient. Values of η_i_ were assumed to be normally distributed with mean 0 and variance ω^2^. Interindividual variances that could not be estimated properly were fixed to 0. In the present study, interindividual variances of kheal, Circ0, MTT and KMAX were estimated, whereas interindividual variances γ, G50 and kel were not estimable and were therefore fixed to 0.

*Residual model*. Additive, proportional and mixed additive-proportional models were tested.

*Covariate model*. The following covariates were tested on model parameters: age at diagnosis, sex, Buchanan score, platelet count at diagnosis, acute/prolonged disease, presence of infections and vaccination within 21 days prior 21 the diagnosis, acute/insidious presentation and platelet count at diagnosis. The influence of potential covariates on structural parameters was assessed in two steps: (1) an univariate step in which the influence of each covariate on model parameters associated with interindividual variability was tested separately. Covariates displaying a significant association with model parameters (α<0.1) were kept for the (2) multivariate step, in which forward and backward stepwise process was made. Covariates displaying a significant association with model parameters when added during forward (α<0.05) and then removed from (α<0.02) covariate model were kept in the final model. Model was developed in the learning subset, i.e. data of 103 patients followed in 3 french centers (Tours, Orléans and Nantes) using Monolix Suite 2020 (Lixoft®, Antony, France).

## Model goodness of fit and evaluation

The goodness-of-fit (2,3) was assessed for each model by plotting individually predicted (IPRED) concentrations versus observed concentrations. Population predictions were obtained using typical parameters, which include explained variability (i.e. population estimates and covariates), whereas individually predicted concentrations were obtained using individual parameters, which include both explained and unexplained (i.e. the random effects η_i_ for each PK parameter). In addition, the goodness-of-fit was evaluated by the distribution of residuals evaluated by graphical inspection of population (PWRES) and individual (IWRES) and normalized prediction distribution errors (NPDE). These residuals should be close to a standard normal distribution (i) to confirm a satisfactory description of the data using the model and (ii) to allow LRT tests (figure 2).

The final model described satisfactorily platelet count over time data (figure 3). Parameters were estimated with good efficacy (table 1). Plots of WRES vs. estimates and NPDE showed no obvious bias or model misspecification (figure 4). Plots of observed vs. predicted platelet counts over time showed satisfactory description of platelet count data. Among tested covariates, initial observed platelet count was found to be significantly inversely correlated with K_MAX_ (p = 2.2 . 10^-16^) and prolonged PTI was significantly associated with decreased k_heal_ value by 70-fold (p = 1.02 . 10^-8^). The best residual model was proportional. Being a first-order rate constant, a «half-life» of recovery may be derived by calculating T½-heal = ln(2)/k_heal_ = 1.6 years in mean in the learning cohort. This half-life of recovery was 6 months and 35 years in acute and prolonged PTI patients, respectively.

**Table S1.** Summary of parameter estimates

| Parameter (unit) | Estimate | RSE (%) | IIV | RSE (%) |
| --- | --- | --- | --- | --- |
| Circ_0_ (G.L^-1^) | 87.5 | 9 | 0.50 | 15.3 |
| MTT (days) | 30 | 30 | 0.37 | 12.1 |
| γ | 0.17 | 4.2 |  |  |
| K_MAX_ (day^-1^) | 5.3 | 8.5 | 0.53 | 8.3 |
| NUM_0__K_MAX_ | -0.07 | 8.6 |  |  |
| G_50_ (─) | 5.4 . 10^-5^ | 16 |  |  |
| k_heal_ (day^-1^) | 3.7 . 10^-3^ | 29 | 2.9 | 9.6 |
| Prolonged PTI_k_heaL_ | -4.2 | 18 |  |  |
| k_el_ (day^-1^) | 1.22 | 0.56 |  |  |
| σ_prop Circ_ (─) | 0.69 | 3.25 |  |  |

**Legends.** RSE: relative standard error, IIV: interindividual variance, kheal: spontaneous disease recovery rate constant, Circ0: baseline platelet count, MTT: mean transit time of platelet maturation, γ: negative feedback exponent, KMAX: maximum disease-induced platelet destruction G50: value of platelet recovery leading to a decrease of K_MAX_ of 50%, and kel: first-order disease persistence rate constant, σ_prop circ_: proportional error standard deviation, NUM_0_, observed platelet count at diagnosis

##
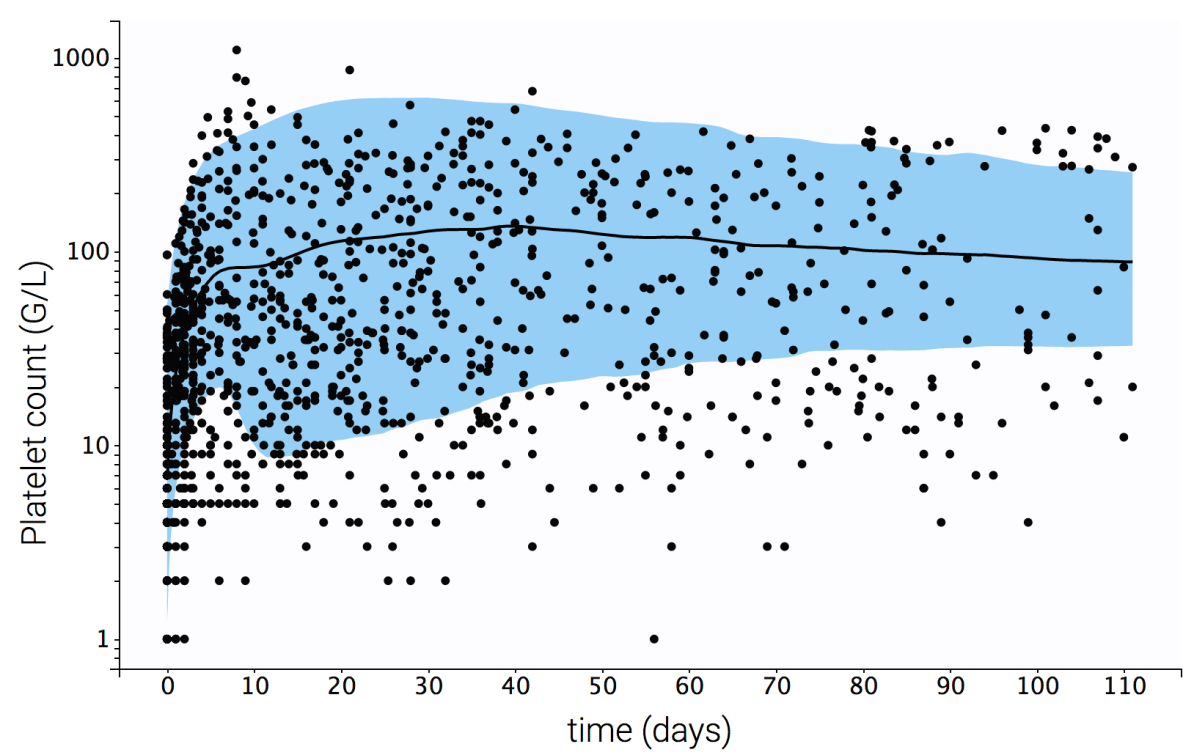


## Figure S3. Observed platelet counts vs. 90% model-prediction interval over time.


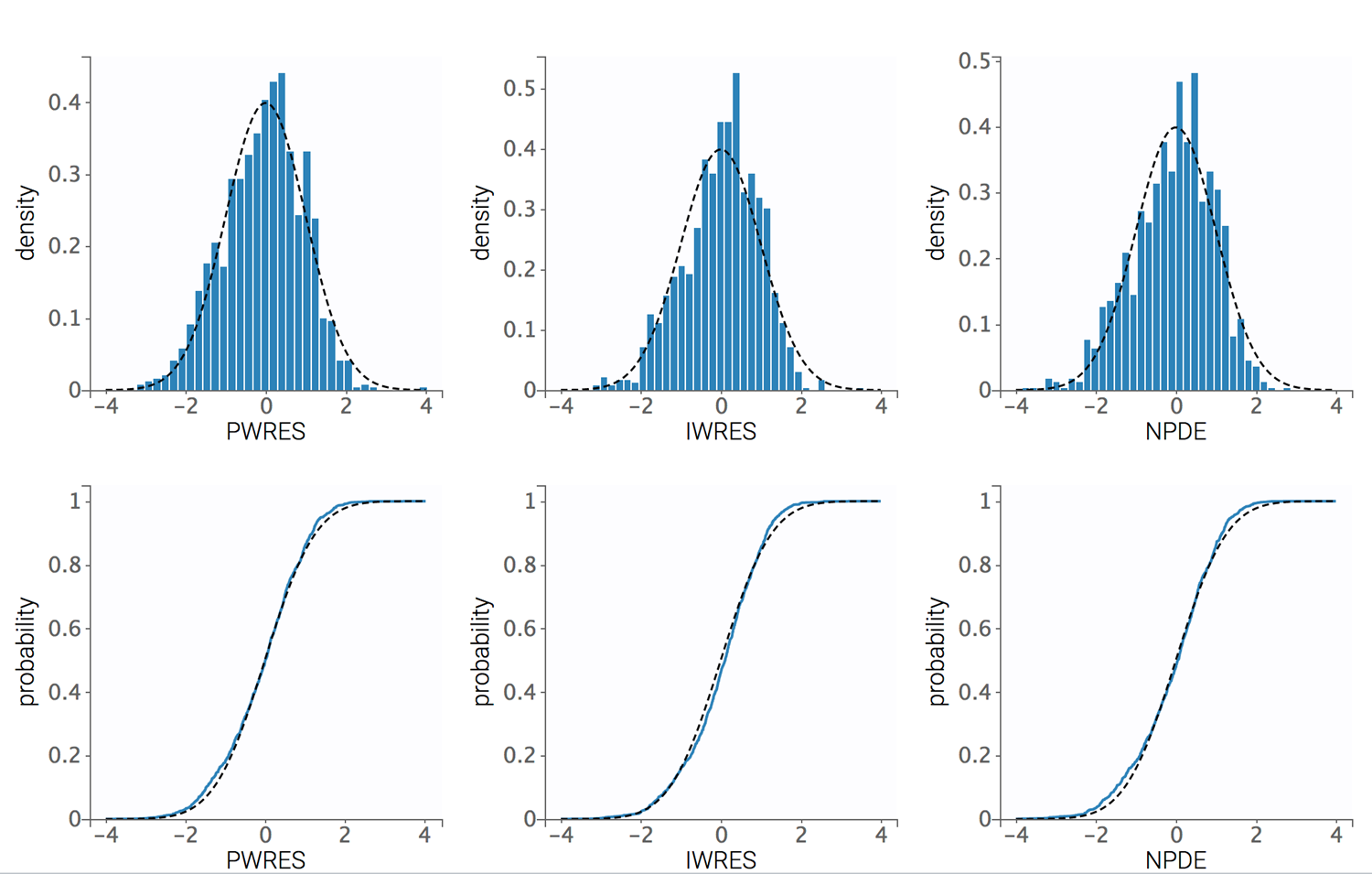


**Figure S4.** Actual (blue) vs. theorical Gauss (black) density (up) and distribution (bottom) functions for population weighted residuals (PWRES, left), individual weighted residuals (IWRES, middle) and normalized prediction distribution error (NPDE, right).

## Model predicting prolonged disease

We showed that prolonged PTI was significantly associated with decreased k_heal_ value by 70-fold (p = 1.02 . 10^-8^). However, the diagnosis of prolonged/acute disease was the clinical endpoint to be predicted, and was made at the end of the follow-up (3 months). Since our model aimed at predicting disease status before the end of follow-up, disease status cannot be used to predict a prolonged disease a covariate. We therefore remove this covariate and use individual k_heal_ estimates to make this prediction. Without prolonged disease covariate, our model results were:

**Table S2.** Summary of parameter estimates of final model

| Parameter (unit) | Estimate | RSE (%) | IIV | RSE (%) |
| --- | --- | --- | --- | --- |
| Circ_0_ (G.L^-1^) | 83.2 | 9.6 | 0.60 | 13 |
| MTT (days) | 31.8 | 6 | 0.40 | 11 |
| γ | 0.21 | 4.2 |  |  |
| K_MAX_ (day^-1^) | 4.8 | 9.3 | 0.54 | 9.2 |
| NUM_0__K_MAX_ | -0.07 | 8.9 |  |  |
| G_50_ (─) | 1.6 . 10^-4^ | 15 |  |  |
| k_heal_ (day^-1^) | 1.2 . 10^-3^ | 31 | 3.0 | 10 |
| k_el_ (day^-1^) | 1.0 | 0.23 |  |  |
| σ_prop Circ_ (─) | 0.69 | 3.25 |  |  |

**Legends.** RSE: relative standard error, IIV: interindividual variance, k_heal_: spontaneous disease recovery rate constant, Circ_0_: baseline platelet count, MTT: mean transit time of platelet maturation, γ: negative feedback exponent, K_MAX_: maximum disease-induced platelet destruction G_50_: value of platelet recovery leading to a decrease of K_MAX_ of 50%, and k_el_: first-order disease persistence rate constant, σ_prop circ_: proportional error standard deviation.

One should keep in mind that parameter estimates were obtained from the learning subset, i.e. 3 French centers (Tours, Orléans and Nantes). Since data from these centers were used to estimate model parameters, they could not be used to demonstrate model predictive performance. We therefore Bayesian analysis to estimate individual parameters of each patient from validation subset, i.e. 2 French centers (Angers and Rennes).

## Bayesian analysis

Bayesian estimation has been used in pharmacokinetics to estimate individual model parameters for limited sampling strategies, especially for immunosuppressive drugs (4). This approach consists in combining:

- prior information about the kinetics of a given compound (mean, interindividual variances and covariate effects), which include prior estimates of kinetic parameters;

- concentration measurements of a given patient for whom individual kinetics have to be estimated. This patient is assumed to belong to the population of interest; in other words, the patient should be similar to the prior population (same disease, drug, etc.).

Bayesian estimation was made on the validation subset. Individual parameters estimates were obtained by fixing interindividual distribution and residual estimates (table 2) as prior values for individual parameter estimates.

Bayesian analysis was applied full validation subset (Vfull) as well as 2 subsets for which validation subset was truncated from platelet counts measured after 30 days (TV30) and 15 days (TV15). Predictive performance of Bayesian models for Vfull, TV30 and TV15 was done by comparing disease evolution forecasting and final diagnosis. These estimates were compared to the threshold value of k_heal_ determined in learning subset: patients with k_heal_ estimates inferior or superior to threshold were inferred as acute or persistent/chronic ITP. Finally, these inferences were compared to actual diagnosis by calculating sensitivity (Se), specificity (Sp), positive (PPV) and negative (NPV) predicting values, and Cohen’s kappa (Ka).

## Upcoming strategies of model improvement

Predictive performance of our model was compared with Schmidt et al.model (5) by calculating disease score and probability using Childhood ITP Recovery Score Calculator (accessed at <https://schmidtdav.shinyapps.io/calc/>). Schmidt model showed higher sensitivity (0.83 vs 0.70) but lower specificity (0.65 vs. 0.75) than using our model (table 3). This may be explained by the fact that Schmidt et al. model was developed on a bigger cohort than ours, which included patients in observation. Their score may therefore bear a higher information level than the distribution of k_heal_ estimated from our cohort. To decipher whether our model could still be improved, we attempted to run our model using schmidt’s score as a covariate of k_heal_ using the strategy presented above. The value of k_heal_ was strongly associated with recovery score (p=2.10^-6^, table 4). In addition, Bayesian estimates of individual k_heal_ in validation subset lead to increased predictive performances (Se=0.86, Sp=0.70, table 4) of our model and of Schmidt’s ITP recovery score (table 4). This suggests that (i) the description of k_heal_ distribution may be improved using a bigger cohort and/or (ii) as stated in discussion, Schmidt et al model and ours might be combined to increase predictive performance of acute vs. persistent/chronic disease.

**Table S3.** Prediction of clinical course in the validation subset using our kinetic model, Schmidt’s ITP recovery score and our kinetic model with score as a covariate on k_heal_ distribution

| **Parameter**  **Validation set** | **kinetic model** | **Schmidt’s ITP recovery score** | **kinetic model with**  **Score as a covariate** |
| --- | --- | --- | --- |
| Sensitivity | 0.70 | 0.83 | 0.86 |
| Specificity | 0.75 | 0.65 | 0.70 |
| PPV | 0.64 | 0.79 | 0.82 |
| NPV | 0.79 | 0.71 | 0.76 |
| Kappa | 0.73 | 0.76 | 0.80 |

Legends. PPV, positive predictive value; NPV, negative predictive value.

**Table S4.** Summary of parameter estimates of final model

| Parameter (unit) | Estimate | RSE (%) | IIV | RSE (%) |
| --- | --- | --- | --- | --- |
| Circ_0_ (G.L^-1^) | 88.6 | 7.9 | 0.50 | 12 |
| MTT (days) | 30 | 5.9 | 0.37 | 12 |
| γ | 0.17 | 1.1 |  |  |
| K_MAX_ (day^-1^) | 5.2 | 13 | 0.51 | 18 |
| NUM_0__K_MAX_ | -0.071 | 11.5 |  |  |
| G_50_ (─) | 3.2 . 10^-4^ | 1.8 |  |  |
| k_heal_ (day^-1^) | 1.1 . 10^-3^ | 30 | 3.0 | 21 |
| **Score on k_heal_** | **29.5** | **25** |  |  |
| k_el_ (day^-1^) | 0.96 | 0.26 |  |  |
| σ_prop Circ_ (─) | 0.69 | 3.9 |  |  |

**Legends.** RSE: relative standard error, IIV: interindividual variance, k_heal_: spontaneous disease recovery rate constant, Circ_0_: baseline platelet count, MTT: mean transit time of platelet maturation, γ: negative feedback exponent, K_MAX_: maximum disease-induced platelet destruction G_50_: value of platelet recovery leading to a decrease of K_MAX_ of 50%, and k_el_: first-order disease persistence rate constant, σ_prop circ_: proportional error standard deviation.

## References

1 Friberg LE, et al. Model of chemotherapy-induced myelosuppression with parameter consistency across drugs. J Clin Oncol. 2002;20:4713-4721.

2 Wojciechowski J et al. Population pharmacokinetic-pharmacodynamic modelling of platelet time-courses following administration of abrocitinib. Br J Clin Pharmacol. 2022;88:3856-3871.

3 Mould DR, Upton RN. Basic concepts in population modeling, simulation, and model-based drug development. CPT Pharmacometrics Syst Pharmacol. 2012 26;1:e6.

4 Mould DR, Upton RN. Basic concepts in population modeling, simulation, and model-based drug development-part 2: introduction to pharmacokinetic modeling methods. CPT Pharmacometrics Syst Pharmacol. 2013;2:e38.

5 Zhao, W. et al. Population pharmacokinetics and Bayesian estimator of mycophenolic acid in children with idiopathic nephrotic syndrome. Br J Clin Pharmacol. 2010:69;358–366.

6 Schmidt DE et al. A clinical prediction score for transient versus persistent childhood immune thrombocytopenia. J Thromb Haemost. 2021;19:121-130.
